# Advances in Understanding Drought Stress Responses in Rice: Molecular Mechanisms of ABA Signaling and Breeding Prospects

**DOI:** 10.3390/genes15121529

**Published:** 2024-11-27

**Authors:** Yingying Ma, Mingyue Tang, Mingyang Wang, Yanchun Yu, Banpu Ruan

**Affiliations:** College of Life and Environmental Sciences, Hangzhou Normal University, Hangzhou 311121, China; 2023111010053@stu.hznu.edu.cn (Y.M.); 2022111010029@stu.hznu.edu.cn (M.T.); 2022111010012@stu.hznu.edu.cn (M.W.); ycyu@hznu.edu.cn (Y.Y.)

**Keywords:** drought stress, rice, signaling pathways, adaptive mechanisms, abscisic acid (ABA)

## Abstract

Drought stress is a pivotal environmental factor impacting rice production and presents a significant challenge to sustainable agriculture worldwide. This review synthesizes the latest research advancements in the regulatory mechanisms and signaling pathways that rice employs in response to drought stress. It elaborates on the adaptive changes and molecular regulatory mechanisms that occur in rice under drought conditions. The review highlights the perception and initial transmission of drought signals, key downstream signaling networks such as the MAPK and Ca^2+^ pathways, and their roles in modulating drought responses. Furthermore, the discussion extends to hormonal signaling, especially the crucial role of abscisic acid (ABA) in drought responses, alongside the identification of drought-resistant genes and the application of gene-editing technologies in enhancing rice drought resilience. Through an in-depth analysis of these drought stress regulatory signaling pathways, this review aims to offer valuable insights and guidance for future rice drought resistance breeding and agricultural production initiatives.

## 1. Introduction

Global food security faces significant challenges due to severe climate change and rapid population growth. By 2030, an estimated 660 million people are expected to still be grappling with hunger [1]. Rice (*Oryza sativa* L.), a major staple for half of the world’s population, is heavily impacted by various abiotic stresses [2]. Drought, in particular, has reduced global rice yields by 25.4% over the past two decades, making it one of the most critical limiting factors [3]. Rice’s water demand during the growing season exceeds that of other crops as paddy fields must remain submerged [4]. By 2025, it is anticipated that 15 to 20 million hectares of irrigated rice fields in Asia will face the threat of continuous drought due to water shortages [5]. Consequently, investigating the regulatory mechanisms and signaling pathways of rice under drought conditions, and enhancing its drought tolerance are essential for maintaining yields and ensuring food security [6].

Drought stress is an abiotic challenge that restricts plant growth and physiological processes due to inadequate water availability. This stress impacts leaf size, stem elongation, and root growth, disrupts water relations within the plant, and decreases water-use efficiency [7], further affecting growth, photosynthesis, ion balance, and seed germination rates, ultimately reducing yields [8]. Rice has evolved various complex adaptive mechanisms [9], including changes at the morphological, physiological, and molecular levels such as gene expression regulation, structural modifications, photosynthesis optimization, increased antioxidant enzyme activity, accumulation of osmotic adjustment substances, and alterations in root morphology and function to counter drought stress [10,11,12,13]. This includes enhancing water absorption capabilities through deeper and more extensive root systems [14], reducing water evaporation by modulating leaf curling and stomatal activity, and maintaining cellular water balance by increasing the content of intracellular osmotic adjustment substances [15]. Additionally, enhancing antioxidant enzyme activity reduces the accumulation of reactive oxygen species, protecting cells from oxidative damage [2]. These adaptive responses involve multiple signaling pathways, including those related to reactive oxygen species (ROS), abscisic acid (ABA), jasmonic acid (JA), calcium, gene expression regulation, and the MAPK cascade [10,11].

Roots serve as the initial detection site for drought signals, which are then relayed to the aerial parts of the plant through complex signaling mechanisms, triggering a series of physiological and molecular responses [16]. Upon drought detection, ABA synthesized in the leaves regulates stomatal closure and optimizes water use efficiency [17]. The transmission of these signals, including ABA, pH changes, and cytokinins, occurs through the vascular system [18]. The adaptation of plants to drought conditions involves significant adjustments in root gene expression and structure, such as alterations in root growth and morphology, to enhance water absorption capabilities [19]. Furthermore, the drought signaling process involves various secondary messengers, including calcium ions (Ca^2^⁺), reactive oxygen species (ROS), and phosphatidylinositol phosphate derivatives [20]. These messengers facilitate signal amplification and transmission, activating downstream signaling pathways such as the mitogen-activated protein kinase (MAPK) and calcium-dependent protein kinase (CDPK) pathways [21], which regulate gene expression and physiological and biochemical responses [22], ultimately leading to the transcriptional activation of drought-responsive genes [23]. These genes help plants cope with drought stress by regulating osmotic adjustment, repairing oxidative damage, and managing growth processes, thereby enhancing plant survival and growth under drought conditions [24]. Understanding the mechanisms of drought signal perception and transmission, as well as the plant’s responses to drought stress, provides a theoretical foundation and technical support for improving drought-resistant plant varieties, boosting plant productivity under drought conditions, and safeguarding global food security [25].

## 2. Adaptation Changes of Rice Under Drought Stress

Rice originally thrived in aquatic environments, retaining physiological and molecular adaptations suitable for water-rich settings [26]. However, as a vital agricultural crop, rice must also adapt to a wide range of field conditions, including drought scenarios [27]. This dual adaptability enables rice to effectively transition between wet and dry conditions swiftly [10]. Exhibiting significant phenotypic plasticity, rice adjusts its morphological and physiological traits in response to environmental variations [28]. For instance, under drought stress, rice may develop a compact form and an enhanced system for water absorption and transportation [29]. Moreover, compared to many terrestrial plants, the rice root system shows greater plasticity under drought conditions [30], swiftly modifying its structure by increasing root length and root hair density to improve water uptake [31]. Drought stress profoundly affects rice across morphological, physiological, biochemical, and molecular dimensions [32].

Morphologically, changes are seen in the root system, leaf architecture, and overall plant stature. Under drought conditions, root length and biomass may decrease, yet some drought-tolerant varieties adapt by enhancing root depth and diameter [33]. Drought also leads to reduced leaf area and size, and diminishes the number and openness of stomata, thereby decreasing transpiration and the efficiency of photosynthesis [13]. Both drought and UVB radiation significantly inhibited the growth of rice and increased the production of reactive oxygen species (ROS) [34]. When exposed to both UVB radiation and drought stress, plant defense response and growth inhibition showed subadditive effects, suggesting a possible cross-resistance mechanism [35]. Furthermore, drought notably reduces the height and total biomass of rice, especially during critical reproductive phases such as flowering and grain filling, where the effects are more severe [36]. Drought adversely impacts pollen viability and anther development, leading to poor grain filling and ultimately reducing yields [37].

Drought not only influences rice’s morphological traits but also initiates a cascade of physiological and biochemical reactions, affecting the plant’s overall health and productivity. Physiologically, the impact of drought stress is characterized by decreased water use efficiency (WUE), altered stomatal regulation, and reduced photosynthetic activity [13]. Water use efficiency is a crucial indicator of a plant’s capacity to maintain growth under arid conditions [38]. In response to drought, rice modulates stomatal conductance to reduce water loss through transpiration, involving complex signaling pathways such as the activation of the abscisic acid (ABA) pathway [39]. Photosynthesis, essential for plant energy conversion and growth, declines under drought due to decreased internal CO_2_ levels, reduced chlorophyll content, and damage to the photosynthetic machinery [36].

Biochemical alterations include a marked increase in the accumulation of osmotic adjustment substances like proline, soluble sugars, and starch in rice [40]. Drought stress also triggers fluctuations in antioxidant enzyme activities and malondialdehyde levels, affecting cellular membrane stability [41]. The antioxidant system, encompassing superoxide dismutase (SOD), catalase (CAT), and glutathione reductase (GR), effectively scavenges reactive oxygen species (ROS), shielding cells from oxidative damage [33]. Moreover, drought modifies metabolic pathways in rice, accumulating primary sugars and most amino acids that function as osmotic regulators to assist the plant in managing drought stress [42].

**Figure 1:**
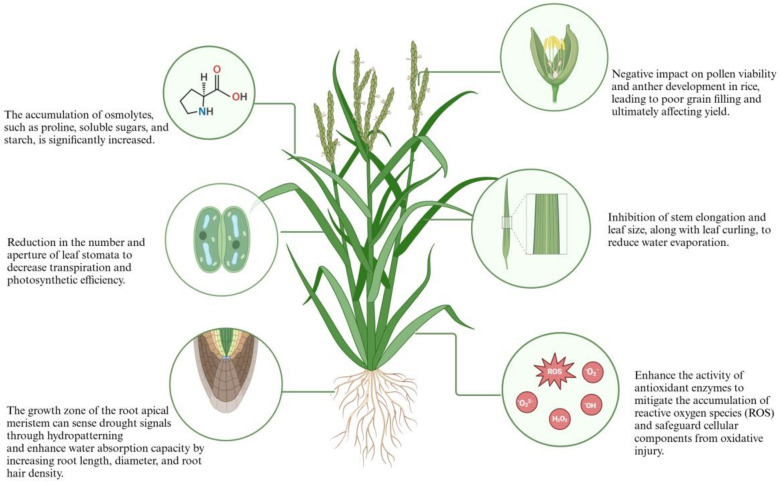
Adaptive changes in rice under drought stress. Under stress induced by aridity, the growth and metabolic processes in rice are attenuated, accompanied by reactive oxygen species (ROS) accumulation. To circumvent cellular injury, rice implements an array of adaptive strategies. These encompass osmotic adjustment to mitigate cellular dehydration, stomatal regulation to optimize water use efficiency, modulation of root architecture to enhance water uptake, activation of antioxidant systems to counteract oxidative stress, adjustment of plant morphological to reduce water loss, and the adoption of precocious maturation as a survival tactic.

On the molecular level, drought induces significant changes in gene expression in rice, activating a variety of signaling pathways [43]. While genes associated with photosynthesis are generally downregulated, those linked to stress resistance are upregulated [44]. Stress-related genes include heat shock proteins (HSPs), late embryogenesis abundant proteins (LEAs), calmodulin-like proteins (CML), and phosphatase 2C (PP2Cs) [11]. Additionally, several transcription factors, such as those from the bZIP, NAC, and MYB families, are activated and play critical roles in responding to drought stress, enhancing rice’s drought tolerance by modulating the expression of downstream genes [13] (Figure 1).

## 3. Perception of Drought Stress Signals

### 3.1. Initial Detection of Drought Signals by Roots

Roots are the first plant organs to detect soil water deficiency, transmitting drought signals to the aboveground parts through sophisticated signaling mechanisms [16]. Under drought conditions, chemical signals originating from the roots are conveyed to the leaves via the microtubule system. These signals include abscisic acid (ABA), changes in pH, cytokinins, ethylene precursors, and malate [45], which collectively work to minimize water loss and slow down leaf growth, thereby modulating the overall drought response of the plant [18]. The growth zones at the root tips are capable of detecting soil moisture distribution through a process known as hydropatterning, which adjusts the positions of lateral root growth accordingly [46]. Upon sensing moisture stress, the roots activate a cascade of responses, synthesizing and releasing various signaling molecules, including plant hormones, peptides, and amino acid derivatives, which are then transported upwards to the shoot via the vascular system [47]. ABA, one of the primary signaling molecules produced in response to moisture stress in roots, travels through the vascular system to the leaves, where it regulates the closure of stomata to reduce water loss [48]. ABA synthesis in the leaves plays a critical role during the early stages of water stress, while synthesis in the roots intensifies in the later stages [49]. Additionally, the long-distance signaling of the small peptide CLE25 produced in the roots also participates in ABA synthesis in the leaves, further regulating ABA accumulation and stomatal control [50]. Stomatal closure is achieved by regulating the activities of ion channels and pumps in the guard cells. The CLE25 peptide is transported upwards to the leaves through the plant’s vascular system and there, it binds to specific receptor proteins, such as BAM1 and BAM3 in the BAM receptor family, triggering downstream signaling pathways, including the activation of the *NCED3* gene, which facilitates ABA synthesis in the leaves [51]. Hydraulic signals triggered by water stress play a crucial role in the signaling process from roots to leaves, preceding both ABA signals and stomatal closure. Overall, when plant roots detect water stress, they activate a series of complex signaling mechanisms. While ABA is one of the main signaling molecules, other chemical signals like cytokinins, ethylene precursors, and small peptides also play critical roles in the signaling process. These molecules and mechanisms collectively regulate the plant’s physiological and biochemical responses to adapt to drought conditions.

### 3.2. Signal Perception at the Cellular Level

Under drought conditions, plants adapt to environmental stress by remodeling and hardening the cell wall [52]. This process involves reactive oxygen species (ROS) and peroxidases, which initially cross-link phenolic compounds and glycoproteins in the cell wall, causing it to harden [53]. Subsequently, sustained elevated levels of ROS lead to the breakdown of polymers, combined with activities of xyloglucan endotransglycosylase/hydrolases (XTH) and expansins, ultimately relaxing the cell wall to promote growth in stressed organs [54]. Changes in cell wall components include increased levels of XTH and expansins, as well as enhanced deposition of hemicellulose and lignin in the secondary wall [55]. These changes help maintain cell turgor and reduce water loss, thereby enhancing the plant’s drought resistance [56]. Changes in the physical state of the cell wall can be detected by mechanical sensors on the cell membrane [57]. Studies have shown that fluid shear stress (FSS) increases membrane fluidity, activating heterotrimeric G proteins, which indicates that the cell membrane serves as an important mechanical sensor [58]. Under drought stress, interactions between the cell wall and the plasma membrane activate receptor-like kinases, cytoskeleton-associated mechanical sensors, stretch-activated ion channels (SACs), and redox-mediated systems [59]. Mechanical sensors such as integrins transmit mechanical signals to the cytoskeleton, regulating the cell’s mechanical response [60]. Mechanosensitive ion channels can rapidly convert mechanical stimuli into biological signals, controlling downstream effector molecules [61]. Receptor-like kinases (RLKs) on the plasma membrane transmit signals internally through their cytoplasmic kinase activity, activating downstream signaling pathways [62]. In summary, under drought stress, mechanical sensors on the cell membrane, along with the activation of receptor kinases, cytoskeleton-associated mechanical sensors, and mechanosensitive ion channels, coordinate with ROS signals, calcium ion signals, and plant hormones to regulate plant growth and metabolism.

### 3.3. Initial Transduction of Drought Signals

**Figure 2:**
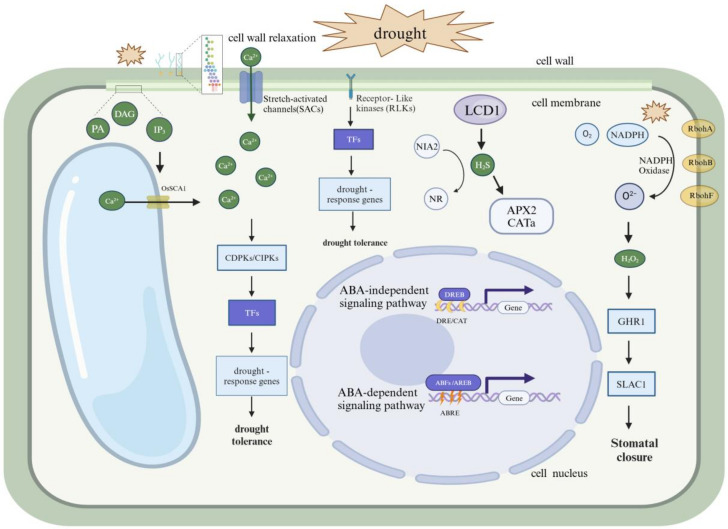
Perception and initial transduction of drought signals at the cellular level. The cell wall perceives signals of water deficiency, leading to a relaxation in its structure. This physical alteration can be detected by mechanosensors on the plasma membrane, triggering the opening of ion channels (SACs). Consequently, Ca^2^⁺ flow inward. Additionally, the breakdown of the plasma membrane generates second messengers such as PA, DAG and IP3. IP3 can bind to Ca^2^⁺ channels on the endoplasmic reticulum or vacuoles, causing the release of Ca^2^⁺ and a sharp change in cytosolic calcium ion concentration. Calcium ions then bind to their specific sensor proteins (e.g., CaM, CDPKs and CBL-CIPK complexes), further activating or inhibiting downstream transcription factors, ultimately leading to changes in the expression of drought stress response genes. NADPH oxidases on the plasma membrane are a primary source of reactive oxygen species (ROS). Under drought stress, NADPH oxidases produce superoxide anions, which are further converted into hydrogen peroxide (H_2_O_2_) signal molecules. H_2_O_2_ can transmit stress signals between cells, activating GHR1, which in turn activates SLAC1. It controls the stomatal aperture through its scaffold function rather than direct phosphorylation of its target proteins [77]. Hydrogen sulfide (H_2_S) is also an important signaling molecule. OsLCD1 can generate H_2_S, which sulfhydrates NIA2, leading to a decrease in NR activity. This process enhances the drought resistance of rice by activating the expression of antioxidant enzyme-encoding genes and ABA-responsive genes [78]. DAG, Diacylglycerol; PA, Phosphatidic Acid; IP3, inositol-1,4,5-triphosphate; CDPK, Calcium-dependent protein kinases; CIPK, Calcineurin B-Like protein Interacting Protein Kinases; TF, Transcription Factor; NIA2, Nitrate Reductase 2; NR, Nitrate Reductase; LCD1, cysteine desulfhydrase; APX2, Ascorbate Peroxidase 2; CAT, Catalase; NADPH Nicotinamide adenine dinucleotide phosphate; GHR1, Guard cell hydrogen peroxide-resistant1; SLAC1, Slow Anion Channel 1; Rboh, Respiratory Burst Oxidase Homolog; DREB, Dehydration Responsive Element Binding protein; DRE, Dehydration Responsive Element; ABRE, Abscisic Acid-Responsive Element.

The cell membrane can detect drought signals and initiate signal transduction through endocytosis and changes in the composition of membrane lipids [63]. Reactive oxygen species (ROS) and abscisic acid (ABA) act as key secondary messengers in the drought response, respectively regulating the redox state and controlling ion channels [64]. Changes in Ca^2^⁺ and pH levels also play critical roles in the transduction of drought signals [65]. These mechanisms work synergistically to help the plant adjust its physiological and biochemical responses under drought conditions, thereby enhancing its drought resistance. Drought stress increases the production of intracellular ROS, which as secondary messengers, trigger adaptive and defensive responses and interact with ABA and Ca^2^⁺ signaling pathways. The generation and scavenging of ROS play a key role in maintaining the cellular redox state, with H_2_O_2_ acting as a secondary messenger in signal transduction [66]. The increase in ABA levels during drought stress promotes stomatal closure through the regulation of ion channels and Ca^2^⁺ signaling pathways, thus minimizing water loss [67]. ABA also plays a crucial role in long-distance signal transduction between roots and leaves, regulating the expression of drought response genes and related metabolic pathways [68]. Ca^2^⁺, as a secondary messenger, is crucial in the transduction of drought stress signals, regulating ion channels and the activity of protein kinases/phosphatases [69]. Changes in pH also participate as signals and messengers in the drought response, regulating intra- and extracellular H⁺ concentrations and related metabolic processes [70] (Figure 2).

It is well known that the cell membrane is primarily composed of a phospholipid bilayer structure made up of glycerol-3-phosphate. Under drought stress, several phospholipases within plant cells are activated, including phospholipase C (PLC), phospholipase D (PLD), and phospholipase A2 (PLA2). These enzymes act on membrane phospholipids to produce various lipid second messengers, such as phosphatidic acid (PA), diacylglycerol (DAG), and inositol triphosphate (IP3) [71]. As second messengers, these molecules can further activate or inhibit other signaling molecules and protein kinases, such as activating protein kinase C (PKC) and mitogen-activated protein kinases (MAPKs) [72]. IP3 can bind to Ca^2^⁺ channels on the endoplasmic reticulum or vacuoles, leading to the release of Ca^2^⁺ from internal stores into the cytoplasm, thus elevating cytoplasmic Ca^2^⁺ concentrations [73]. The increased Ca^2^⁺ levels activate calmodulin-dependent protein kinases (CDPKs) and other kinases, which in turn trigger various downstream responses [74]. Like its associated reactive oxygen species, the phospholipid signaling system acts as a double-edged sword: at low levels, it may activate adaptive responses, while at high levels, it may indicate stress damage or be potentially harmful [75]. Ultimately, these signaling pathways influence the nucleus by activating or inhibiting specific transcription factors, altering gene expression patterns in response to environmental changes [76] (Figure 2).

## 4. ABA-Dependent Signaling Pathways

### 4.1. Basic Pathways of ABA Signaling

In rice, ABA is a key phytohormone that regulates the drought stress response by modulating stomatal closure, root structure, soil microbial community organization, gene expression, and metabolic pathways [67]. Despite the revealed key role of ABA in plant drought resistance, the mechanisms of ABA signaling in monocots like rice remain largely unexplored. Under drought conditions, the synthesis of ABA in plants increases, primarily due to the upregulated expression of ABA synthesis genes such as *NCED*, *ABA2*, and *ABA1* [79]. The synthesis and action of ABA involve multiple biosynthesis sites and transport proteins, forming a complex signaling network [80]. ABA signaling primarily occurs through a signaling module comprising PYR/RCAR receptors, PP2Cs, and SnRK2s, which interact with targets such as ion channels and transcription factors to regulate ABA-induced responses. ABA initiates signaling by binding to its receptors, such as the PYR/PYL/RCAR protein family [81]. PP2Cs inhibit the activity of SnRK2 kinases, but the binding of ABA to PYR/PYL/RCAR receptors inhibits PP2Cs’ activity, thereby activating SnRK2 kinases. Activated SnRK2 kinases further phosphorylate specific transcription factors, thus regulating the expression of downstream genes [82].

### 4.2. ABA Receptors

**Figure 3:**
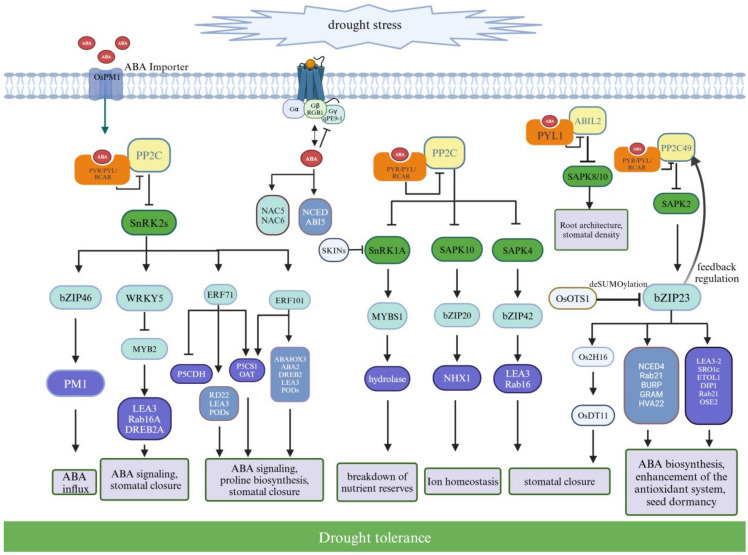
ABA-mediated signaling pathways under drought stress. During conditions of drought stress, ABA functions as a key signaling molecule, initiating signal transduction processes within and between cells. The ABA receptors, which are part of the PYR/PYL/RCAR protein family, can suppress the activity of PP2Cs, thus lifting the inhibition imposed on SnRK2s. The subsequent activation of SnRK2s triggers a cascade of downstream signaling events, including the activation of ion channels and transcription factors. This leads to the transcriptional regulation of a network of genes implicated in the ABA response and the response to drought stress. The signaling network mediated by ABA is crucial for orchestrating physiological responses such as the biosynthesis of ABA, osmotic adjustment, maintenance of ionic balance, regulation of stomatal aperture, activation of antioxidant mechanisms, and modulation of growth and metabolic processes. In the schematic representation, the orange figures denote the PYR/PYL/RCAR family of ABA receptors, the yellow figures represent PP2Cs, the green figures symbolize SnRK2s, the cyan figures correspond to transcription factors, the purple figures indicate genes specifically responsive to drought stress, and the blue figures refer to genes involved in the ABA-dependent response. ABA, Abscisic Acid; PYR/PYL/RCAR, Pyrabactin Resistance 1/PYR1-Like/Regulatory Component of ABA Receptor; SnRK, Sucrose nonfermenting-1-related protein kinase; Gα, G protein α subunit; Gβ, G protein β subunit; Gγ, G protein γ subunit; RGB1, Rice Heterotrimeric G protein β subunit 1; qPE9-1, Quantitative Trait Locus for Panicle Erectness 9-1; NCED, 9-cis-epoxycarotenoid dioxygenase; SAPK, Stress-activated protein kinases; PYL1, Pyrabactin Resistance 1-like 1; ABIL2, Abscisic Acid-insensitive Like2; PP2C, Protein Phosphatase 2C.

The ABA signaling pathway in rice, which regulates drought stress, involves multiple ABA receptors and regulatory factors. For instance, receptors of the *OsPYL/RCAR* family (such as *OsPYL1*, *OsPYL6*, *OsPYL9*, *OsPYL10*) play significant roles in ABA signaling and drought tolerance. Additional receptors and regulatory factors like *OsPSKR15* and OsABAR1 also enhance rice’s drought tolerance through various mechanisms. The interaction between *OsPYL1* and *OsABIL2* activates *SAPK8* and *SAPK10*, regulating ABA signaling and root development [83]. *OsPYL/RCAR7* interacts strongly with *OsPP2CAs* under high ABA concentrations, and although its signaling activity is low, it enhances drought tolerance [84]. *OsPYL6* enhances drought tolerance by regulating ABA accumulation and the expression of drought response genes, though it may reduce yield under non-stress conditions [85]. Editing *OsPYL9* with CRISPR/Cas9 enhances drought tolerance and yield, regulating circadian rhythms and stress proteins [83,86]. Overexpression of *OsPYL10* enhances drought stress tolerance by regulating ABA accumulation and the expression of drought-related genes [87]. Additionally, as a positive regulator of ABA signaling, *OsPSKR15* enhances drought tolerance by interacting with *OsPYL11* [88]. *OsABAR1* enhances drought and salt stress tolerance by regulating ABA metabolism genes and response genes [89] (Figure 3).

### 4.3. Protein Phosphatase 2C Family (PP2Cs)

Protein phosphatase 2C (PP2C) serves as a key negative regulator in the ABA signaling pathway, with known functional members including *OsPP2C09*, *OsPP108*, *OsPP18*, and *OsABIL2*. *OsPP2C09* functions in both ABA-dependent and independent stress signaling pathways, and its overexpression makes plants more sensitive to osmotic stress. *OsPP2C09* interacts with core components of the ABA signaling pathway, inhibiting ABA signals, thereby promoting root growth under drought stress [90]. Additionally, *OsPP2C09* can positively regulate drought responses by directly interacting with DRE-binding proteins and activating the expression of genes with DRE elements in their promoters [91]. *OsPP108* is highly induced under ABA, salt, and drought stress, and Arabidopsis overexpressing *OsPP108* exhibits insensitivity to ABA and tolerance to high salt, mannitol, and drought stress [92]. *OsPP18* enhances rice’s osmotic and oxidative stress tolerance by regulating reactive oxygen species (ROS) balance; its expression is induced by drought stress but not by ABA [93]. *OsABIL2* acts as a negative regulator in ABA signaling, can inhibit the activity of SAPK8 and SAPK10, and overexpression of *OsABIL2* significantly alters plant developmental phenotypes, including stomatal density and root structure, leading to sensitivity to drought stress [94] (Figure 3).

### 4.4. SNF1-Related Protein Kinase 2 Family (SnRK2s)

SnRK2 kinases phosphorylate key components of the stomatal complex, such as the slow anion channels SLAC1 and SLAC7, to induce stomatal closure. This mechanism effectively reduces water transpiration and enhances plant water use efficiency [78]. Known SnRK2 kinases involved in drought stress response include SAPK2, SAPK4, SAPK9, SAPK10, and SnRK1A. SAPK2 activates the transcription factor bZIP23, which then regulates the expression of the ABA synthesis gene *OsNCED4* and drought-responsive genes such as *OsLEA3-2*, *OsSRO1c*, and *OsETOL1* [95], orchestrating seed dormancy, stomatal closure, and the antioxidant system to enhance rice’s drought tolerance. *OsbZIP23* directly increases transcription levels of the inhibitory factor *OsPP2C49*, facilitating ABA level feedback regulation [96].

The small peptide *OsOTS1*, by deSUMOylating *OsbZIP23*, negatively regulates its downstream drought-resistant genes [97]. The expression of *OsDT11*, regulated by multiple signaling pathways, is enhanced by overexpression, which activates bZIP23 and subsequently upregulates ABA-responsive genes (such as *BURP*, *GRAM*, and *HVA22*) and drought-responsive genes (like *DIP1*, *Rab21*, and *OSE2*), effectively regulating ABA concentration and optimizing stomatal density to boost rice’s drought resistance [97]. SAPK4 activates *OsbZIP42*, which has undergone protein modification. *OsbZIP42* suppresses *PP2C* genes (like *ABI2*) and activates downstream ABA-responsive genes *LEA3* and *Rab16*, improving rice’s drought resistance [98]. SAPK9 enhances rice’s drought tolerance and yield by modulating cell osmotic potential, closing stomata, and regulating the expression of stress-responsive genes [99].

The *SAPK10-bZIP20-NHX1* module is an important module for improving plant drought resistance. Under drought conditions, SAPK10 phosphorylates and activates bZIP20, boosting its binding affinity to the ABRE elements in the promoter of the *NHX1* gene, a K^+^-Na^+^/H^+^ antiporter located on the vacuolar membrane, thereby activating *NHX1* expression and enhancing plants’ tolerance to drought and salt stress [100]. Moreover, SnRK1A plays a critical regulatory role in source-sink communication, responding to nutritional demand signals from germinating embryos and seedlings by facilitating nuclear localization of SnRK1A and MYBS1, which in turn triggers the expression of α-amylase and other hydrolytic enzymes essential for mobilizing nutrients in the endosperm. Drought strongly induces the expression of its negative regulator *SKINs*, potentially causing SnRK1A to move from the nucleus to the cytoplasm, inhibiting its ability to activate hydrolytic enzymes [101]. In conclusion, SnRK2s in rice play a pivotal role in regulating drought stress and ABA signaling pathways. Through interactions with other signaling molecules and transcription factors, they govern the expression of multiple drought-resistant genes, offering significant gene candidates for future crop improvement research (Figure 3).

### 4.5. ABA Signaling Modules and Their Functions

ABA signaling modules in rice consist of *OsPYL/RCAR5*, *OsPP2C30*, *SAPK2*, and *OREB1* that regulates ABA-responsive gene expression during seed germination and early seedling growth [102]. The ABA receptor *OsPYL/RCAR7* requires a high concentration of ABA to interact with *OsPP2CAs*. Overexpressing *OsPYL*/*RCAR7* not only enhances drought tolerance but also avoids growth defects [84]. ABA activates the G protein’s Gβ subunit, RGB1, and reduces the activity of the Gγ subunit, qPE9-1, thereby inducing the expression of transcription factors like *OsABI5*, *OsNAC5*, and *OsNAC6*. These transcription factors bind to specific cis-elements, such as ABRE, regulating the expression of downstream stress-responsive genes, promoting ABA biosynthesis, and adjusting rice’s drought adaptability [103].

Activation of the AP2/ERF family of transcription factors through the ABA signaling pathway plays a significant role in drought stress regulation, exemplified by *OsERF71* and *OsERF101*. Overexpression of *OsERF71* in transgenic rice upregulates genes associated with proline synthesis, such as *OsP5CS1* and *OsOAT*, and downregulates proline degradation genes, such as *OsP5CDH*. It also significantly increases the expression of ABA-responsive genes (like *RD22*, *LEA3*, *PODs*) and drought-responsive genes (such as *ABA8OX3*, *ABA2*, *DREB2*, *LEA3*, *P5CS1*, *OAT*, and *PODs*), substantially enhancing rice’s drought tolerance [104]. OsERF71 also enhances drought tolerance by increasing the expression of ABA response and proline biosynthesis genes. Li et al., cloned the *OsERF71* gene from the drought-tolerant rice variety IRAT109, discovering that this gene regulates several ABA-responsive genes (like *OsAB15*, *OsPP2C68*, *OsRab16C*, *OsRab16D*) and proline biosynthesis genes (like *OsP5CS1* and *OsP5CS2*) through an ABA-dependent pathway, increasing proline accumulation and significantly enhancing rice’s drought tolerance [105].

*OsWRKY5* binds directly to the W-box sequence in the *OsMYB2* promoter, suppressing *OsMYB2* expression and thereby downregulating the transcription of its downstream genes (like *OsLEA3*, *OsRAB16A*, and *OsDREB2A*). This regulatory mechanism decreases the drought tolerance of rice [106]. These studies not only demonstrate the complexity of the ABA signaling network but also reveal the mechanisms by which plants finely regulate multiple signaling molecules and transcription factors to respond to environmental stresses, providing essential theoretical foundations and strategic directions for future genetic engineering of crops to enhance drought tolerance (Figure 3).

## 5. ABA-Independent Pathways in Drought Response

### 5.1. ROS Signaling Pathway

Drought stress often leads to excessive accumulation of reactive oxygen species (ROS) [107]. As signaling molecules, ROS play dual roles in plants’ stress response and adaptation processes [108]. Adequate ROS can enhance plants’ resilience by modulating signal transduction pathways, whereas excessive ROS can cause oxidative stress, leading to cellular damage and programmed cell death (PCD). For instance, *OsSPL10*, a member of the Squamosa Promoter-Binding Protein-Like (SPL) family, regulates the expression of *OsNAC2*, *OsAP37*, and *OsCOX11*, reducing ROS accumulation and preventing PCD [109]. Knockdown or knockout of *OsSPL10* facilitates rapid stomatal closure, preventing water loss and enhancing drought resistance. Additionally, genes such as *OsRbohA* [110], *OsRbohB* [111], and *OsRbohF* [112], encoding NADPH oxidases, enhance rice drought tolerance by regulating ROS production and the ABA signaling pathway under drought conditions. *DSM1*, a Raf-like MAPKKK gene, plays a significant role in early signal transduction and enhances drought tolerance by clearing ROS [113]. *OsADR3*, a C2H2-type transcription factor, boosts drought tolerance by inducing antioxidant defense mechanisms and regulating *OsGPX1* expression [114]. *OsGRXS17* participates in the drought response by controlling the concentration of H2O2 in guard cells and stomatal closure, with rice showing lower rates of water loss and stomatal conductance when this gene is downregulated [115]. *OsPP18*, a PP2C family gene, negatively regulates rice’s tolerance to drought and oxidative stress through an ABA-independent ROS clearance pathway [93]. *OsHSP50.2*, a member of the Heat Shock Protein 90 family, is upregulated during heat and osmotic stress, significantly enhancing rice’s tolerance to drought and osmotic stress [116]. Together, these mechanisms regulate ROS clearance in rice under drought stress through a complex signaling network, thereby mitigating oxidative damage. A deeper understanding of these key factors and their interactions is crucial for breeding more drought-resistant rice varieties.

### 5.2. Calcium Signaling Pathway

Ca^2^⁺ serves as a crucial second messenger in plants, involved in responses to various environmental stimuli [117]. During drought stress, intracellular Ca^2^⁺ levels change rapidly, sensed and transduced by calcium-dependent protein kinases (CDPKs) and other calcium-binding proteins, which then induce the expression of downstream stress-responsive genes [118]. Several CDPKs are pivotal in the drought response in rice [119]. For example, overexpression of *OsCDPK7* significantly enhances tolerance to drought and salt stress by boosting the expression of certain stress-responsive genes [120]. Similarly, *OsCPK9* improves plants’ drought tolerance and spikelet fertility by enhancing stomatal closure and osmotic regulation [121]. *OsCPK4* protects cell membranes from lipid peroxidation and reduces electrolyte leakage under drought and salt stress, thus enhancing tolerance [122]. Beyond CDPKs, CBL-interacting protein kinases (CIPKs) also play significant roles in drought stress regulation, like *OsCIPK17*, which enhances drought tolerance by regulating citric acid metabolism in the TCA cycle [123]. *OsANN3*, a calcium-dependent lipid-binding annexin, increases drought tolerance by modulating the ABA signaling pathway, promoting stomatal closure, and reducing water loss [124]. The calcium signaling pathway significantly interacts with other pathways, such as the ABA pathway and processes involved in energy metabolism, photosynthesis, and cell wall metabolism, collectively modulating the physiological and molecular responses of plants to drought [125]. Specific oscillation patterns (calcium signatures) activate a series of Ca^2^⁺-binding proteins and downstream effectors (such as CDPKs, CIPKs, and annexins), transmitting stress signals and regulating the expression of specific response genes [126]. Manipulating the expression of key regulators in the calcium signaling pathway, such as various CDPKs and CIPKs, can significantly enhance rice’s drought tolerance, providing theoretical and practical guidance for improving crop drought resistance through genetic engineering.

### 5.3. MAPK Signaling Pathway

The Mitogen-Activated Protein Kinase (MAPK) signaling pathway is central to plants’ responses to environmental stresses [127]. The MAPK cascade involves MAPKKKs (MAPK kinase kinases), MAPKKs (MAPK kinases), and MAPKs. As the most upstream components, MAPKKKs encode kinases that respond to environmental signals [128]. In rice, the Raf-like MAPKKK gene *DSM1* enhances drought tolerance by regulating ROS clearance. *OsEDR1*, another significantly induced MAPKKK gene, performs exceptionally under drought and salt stress [129]. In Arabidopsis, MAPKKK18 regulates drought tolerance by activating its downstream MAPKK3, a mechanism likely similar in rice. MAPKKs serve as bridge components in the cascade, activating downstream *MPK6* and *MPK3*, enhancing rice’s drought tolerance and disease resistance [130]. MAPKs directly participate in regulating the expression of stress-response genes, such as overexpression of *OsMAPK5* significantly enhancing rice’s tolerance to drought, salt, and cold stresses [131]. Overexpression of *OsMAPK33* increases sensitivity to salt stress, indicating its negative regulatory role in ion homeostasis [132]. The MAPK signaling pathway regulates the expression of downstream genes in response to drought stress by phosphorylating transcription factors [133]. For instance, the *OsWRKY30* transcription factor is activated by phosphorylation by several MAPKs, enhancing rice’s drought tolerance. Additionally, MAPK phosphatases (MKPs) negatively regulate the pathway by dephosphorylating MAPKs, such as overexpression of *OsIBR5* increasing the sensitivity of transgenic tobacco to drought and H2O2 treatment [134]. Overall, the MAPK signaling pathway regulates rice’s response to drought stress through complex interactions among its components, transcription factors, and MAPK phosphatases, with different components helping rice cope with drought stress through various mechanisms, such as regulating ROS clearance, activating downstream transcription factors, and altering ion homeostasis.

## 6. Regulation by Other Plant Hormones

### 6.1. Role of Jasmonic Acid in Drought Stress Regulation in Rice

Jasmonic acid (JA) is pivotal in plants’ response and adaptation to various abiotic stresses including drought, salinity, and cold. During drought stress, JA levels significantly rise, enhancing plants’ drought tolerance through the modulation of gene expression and signaling pathways. A key component in the JA signaling pathway is the transcription factor *OsbHLH148*, which boosts rice drought tolerance by upregulating the expression of *DREB* and *JAZ* genes [135]. Furthermore, transcription factor WRKY13 manages the cross-talk between drought and disease resistance by selectively binding different cis-elements. In the responses to drought and bacterial infection, WRKY13 inhibits the drought-associated gene *SNAC1* and regulates its own expression via specific cis-elements [136]. Additionally, JA exhibits both synergistic and antagonistic interactions with other plant hormones like ABA [137]. For example, the JA and ABA signaling are integrated through the *JAZ-MYC* module, coordinating additional transcription factors and genes to regulate the response mechanisms in rice [138]. Research indicates that overexpression of *OsJAZ9* increases levels of ABA and JA, thus improving drought resistance in rice by regulating leaf width and stomatal density [139]. Also, *OsJAZ1*, as a negative regulator in the JA signaling pathway, suppresses gene expression within the JA and ABA pathways, diminishing rice’s drought resistance [140]. Similarly, *OsPUB16*, another negative regulator within the JA pathway, decreases rice’s drought resistance by controlling the ‘*SAPK9-OsMADS23-OsAOC*’ module, which suppresses the synthesis of ABA and JA [141]. Interestingly, the *cpm2* mutant, lacking the JA synthesis-related gene *AOC*, exhibits enhanced drought resistance, suggesting that JA might negatively regulate drought responses under certain conditions [142].

### 6.2. Role of Ethylene in Drought Stress Regulation in Rice

Ethylene is a critical plant hormone that significantly influences plant growth, development, and response to environmental stresses. The ethylene signaling pathway’s specific mechanisms and pathways are a focus of research in drought stress regulation in rice. Ethylene signal transduction in rice involves several conserved signaling components such as EIN2, EIN3, and CTR1, which also perform similarly in *Arabidopsis* [143]. *OsEIN2* and *OsEIL1* are crucial regulatory factors in ethylene signal transduction that govern growth and stress responses in rice [144]. *OsDERF1*, an important ethylene response factor (ERF), negatively regulates ethylene-mediated drought responses by binding to the GCC box of ERF inhibitors such as *OsERF3* and *OsAP2-39*, thus inhibiting ethylene synthesis and reducing drought tolerance in rice [145]. The expression of AP2/ERF transcription factors, regulated by ethylene signals, modulates stress-related gene expression, affecting rice’s adaptability to drought [146]. Ethylene maintains its synthesis at high levels under stress by regulating key biosynthesis genes (e.g., *OsACS5*, *OsACO1*, *OsACO3*) and signaling components (e.g., *EIN2*, *EIL1*), facilitating physiological and biochemical adjustments in plants under drought stress and enhancing drought resistance [147]. Moreover, interactions between ethylene and other hormones like gibberellins and abscisic acid are crucial for plant growth regulation under drought stress [148]. Ethylene balances gibberellins and abscisic acid to promote internodal growth and the formation of aerial roots in rice under flooded conditions [149]. It also enhances disease resistance in rice by interacting with jasmonic acid (JA) and reactive oxygen species (ROS) signaling pathways [150].

### 6.3. Role of Auxin in Drought Stress Regulation in Rice

Auxin plays a complex and critical role in rice’s response to drought stress, primarily through the precise regulation of its signaling, transport, and metabolism. In terms of signaling, members of the Aux/IAA gene family, such as *OsIAA6* and *OsIAA20*, are significantly upregulated under drought stress. These genes enhance drought tolerance by modulating auxin biosynthesis genes or ABA signaling pathways [151,152]. Additionally, auxin response factors (ARFs) contribute to rice’s adaptation to drought and salt stresses by influencing soluble sugar content, root development, and chlorophyll levels, aiding the plant in overcoming environmental challenges [153].

In auxin transport, members of the PIN gene family, such as *OsPIN3t* and *OsPIN1b*, exhibit altered expression and subcellular localization under drought conditions. Overexpression of *OsPIN3t* enhances drought tolerance, while loss of function in *OsPIN1b* reduces it [154,155]. On the metabolic front, drought stress reduces endogenous auxin levels in rice panicles. However, exogenous auxin application can mitigate the adverse effects of drought on pollen viability, panicle seed-setting rate, and yield [156]. Moreover, drought stress downregulates the expression of auxin biosynthesis genes such as *OsYUCCA*, leading to lower endogenous auxin levels, which in turn impairs reproductive development and drought resilience [157].

Beyond direct hormonal actions, transposons like INDITTO2 enhance drought tolerance by promoting root growth through the regulation of DRO1 gene expression [158]. Collectively, auxin enhances rice drought tolerance by regulating Aux/IAA and ARF signaling, PIN-mediated transport, and its biosynthesis and metabolism. These insights provide a theoretical foundation and practical strategies for breeding drought-tolerant rice.

### 6.4. Role of Gibberellic Acid in Drought Stress Regulation in Rice

Gibberellic acid (GA) plays a pivotal role in rice’s response to drought stress through its interaction with abscisic acid (ABA) and coordinated regulation of stress responses. Under drought conditions, changes in GA metabolism reduce GA levels, leading to suppressed plant growth. This reduction in GA promotes the accumulation of the signaling repressor SLENDER RICE 1 (SLR1), which competitively binds to the coactivator TAD1, stabilizing the ABA receptor PYL10 and enhancing ABA signaling. This synergistic interaction improves drought tolerance [159].

The soluble GA receptor GID1 is essential in regulating stomatal development and function. Under drought conditions, gid1 mutants show impaired endogenous ABA biosynthesis but heightened sensitivity to exogenous ABA, highlighting GID1’s role in ABA-GA cross-talk [160].

During seed germination, GA3 significantly improves the vigor, root length, and shoot length of drought-resistant rice seeds by upregulating genes involved in α-amylase and expansin synthesis [161]. GA also works synergistically with heme oxygenase-1 (HO-1) to regulate programmed cell death in the aleurone layer, facilitating seed germination [162].

Furthermore, transcription factors like ZFP185 influence GA and ABA levels, thereby affecting rice growth and stress responses. Overexpression of ZFP185 decreases GA3 levels, resulting in a semi-dwarf phenotype and heightened sensitivity to drought, cold, and salt stresses [163]. Similarly, *OsCYP71D8L* modulates the balance between GA and cytokinin, and its overexpression confers a dwarf phenotype with enhanced drought and salt tolerance [164].

In summary, GA contributes to rice drought tolerance through mechanisms including synergistic regulation with ABA, seed germination control, and modulation of growth and stress responses. These mechanisms provide valuable genetic resources and theoretical guidance for breeding drought-resistant rice.

### 6.5. Role of Salicylic Acid in Drought Stress Regulation in Rice

Salicylic acid (SA), a key plant hormone, plays a crucial role in enhancing rice drought tolerance through multiple mechanisms. First, SA significantly boosts the activity of antioxidant enzymes, such as catalase and superoxide dismutase, which scavenge reactive oxygen species (ROS) and reduce lipid peroxidation. This protects cell membranes from oxidative damage [165].

Second, SA enhances drought tolerance by regulating the expression of genes involved in antioxidant defense and osmotic adjustment. It promotes the accumulation of osmolytes such as proline, soluble sugars, and starch, which are vital for maintaining cellular osmotic balance and alleviating osmotic stress [166,167].

SA also improves water use efficiency (WUE) by increasing leaf relative water content (RWC), reducing transpiration rates, and maintaining stomatal conductance and photosynthetic efficiency [168]. Additionally, SA enhances photosynthetic pigment levels, promoting photosynthesis and biomass accumulation [169]. Furthermore, SA improves the uptake and utilization of nutrients such as nitrogen, phosphorus, and potassium, further supporting plant growth under drought stress [170].

In summary, SA enhances rice drought tolerance by boosting antioxidant defenses, promoting osmolyte accumulation, improving WUE, and enhancing photosynthesis. These features make SA a promising regulator for mitigating drought stress in rice.

### 6.6. Role of Brassinosteroids in Drought Stress Regulation in Rice

Brassinosteroids (BRs) play an essential role in rice’s drought stress responses by fine-tuning key regulatory factors and signaling cross-talk to enhance stress tolerance. The kinase GSK3/SHAGGY-like kinase 2 (GSK2), a negative regulator of BR signaling, phosphorylates the BR-positive regulator DLT. This interaction enhances BR signaling, alleviating drought-induced phenotypes [171].

The BR receptor BRI1 and its homolog BdBRI1 are also critical for drought response. Downregulation of BdBRI1 results in a dwarf phenotype with improved drought tolerance [172]. The transcription factor *OsNAC016* operates at the intersection of BR and ABA signaling, interacting with GSK2 and SAPK8 to regulate both BR-mediated growth responses and ABA-mediated drought adaptations [173].

BRs also interact antagonistically with ABA, co-regulating critical drought-response genes. Transcription factors such as *OsNAC016* and *RD26* play key roles in balancing growth and drought tolerance through BR-ABA pathways [174,175]. Additionally, *OsSHI1* coordinates multiple hormonal pathways, including BR, ABA, and auxin. It enhances drought tolerance by activating BR and auxin biosynthesis while suppressing ABA signaling [157].

In conclusion, BRs significantly improve rice drought tolerance through mechanisms such as optimizing water use efficiency, enhancing antioxidant systems, regulating gene expression, and promoting growth and metabolism. These capabilities establish BRs as a powerful tool for developing drought-tolerant rice varieties.

### 6.7. Role of Strigolactones in Drought Stress Regulation in Rice

Strigolactones (SLs) play a significant role in the drought resistance mechanisms of rice. They modulate the plant’s response to drought stress through various mechanisms, including interaction with abscisic acid (ABA), regulation of stomatal closure, promotion of antioxidant defense, and interaction with root-associated microbes [176]. Studies have demonstrated a notable interplay between SLs and ABA in the drought response of rice [177]. Under drought conditions, the biosynthesis of SLs in the roots and ABA in the stems of rice, along with the expression of SLs biosynthetic genes, are concurrently induced [178]. SLs biosynthetic mutants, such as *d10*, *d17*, and *d3*, exhibit higher ABA concentrations under drought conditions compared to wild-type plants and show greater drought tolerance [179]. Furthermore, strigolactones can enhance a plant’s tolerance to stress by regulating reactive oxygen species within the plant, increasing the involvement of protective substances in plant osmotic regulation, and improving photosynthesis, thereby enhancing the plant’s resilience to adverse conditions [180]. Under drought stress, the SLs’ biosynthetic and signaling pathways play a crucial role in plant drought resistance, and the overexpression of genes in the SLs’ biosynthesis and signaling pathways can significantly improve root drought tolerance [181].

## 7. Gene Expression Regulation

### 7.1. Regulatory Mechanisms of Key Transcription Factors

Rice plants respond to abiotic stresses such as drought by employing complex signaling pathways to adjust their physiological and developmental processes. Central to the regulation of these pathways are transcription factors [182]. These factors control the expression of relevant genes, thereby initiating mechanisms of adaptation and resistance. For example, *OsNAC022*, a stress-responsive NAC transcription factor, is significantly upregulated under conditions of drought, high salinity, and abscisic acid (ABA) induction. It initiates transcription activation responses by binding to specific cis-elements, thus enhancing plants’ tolerance to drought and salt stress [183]. Likewise, *OsNAC016*, another crucial NAC transcription factor, affects plant structure and stress tolerance by regulating the synthesis of brassinosteroids (BR) and ABA responses, playing a vital role in rice growth, development, and drought regulation [173]. Additionally, the WRKY family transcription factor WRKY13 manages the crosstalk between abiotic and biotic stress signaling pathways (such as drought and disease) by selectively binding to different cis-elements. It regulates antagonistic interactions between drought and disease resistance pathways by suppressing the expression of *SNAC1* and *WRKY45-1* genes [184]. *OsWRKY45*, another key member of the WRKY family, exhibits significant expression under ABA and various stress factors like salt, drought, and temperature changes, enhancing the drought and disease resistance traits of rice [185]. Similarly, *OsbZIP23*, a pivotal bZIP transcription factor, governs ABA signaling and biosynthesis under drought stress, playing a central role in rice drought resilience [98]. *OsbZIP46*, akin to other bZIP transcription factors such as *OsbZIP23*, manages ABA-mediated drought responses by binding to AREB elements. *OsNF-YA7* controls drought tolerance through an ABA-independent mechanism. Its promoter region contains three ABA-independent DRE/CTR elements, and its expression during drought stress is not dependent on ABA, unlike *OsNF-YA4*, which is significantly influenced by ABA, demonstrating different regulatory mechanisms in rice drought response [186]. *OsADR3*, a C2H2-type zinc finger transcription factor, enhances plants’ ROS scavenging capabilities by regulating the expression of the antioxidant enzyme gene *OsGPX1*, thereby strengthening ABA-mediated responses and ultimately improving plants’ drought tolerance [114]. *OsMYB48-1*, a MYB-related transcription factor, enhances the survival of rice under drought conditions by regulating the expression of ABA biosynthesis and signaling genes [187]. Additionally, *OsNAC006* is a key NAC transcription factor, whose mechanism in regulating drought response is not entirely understood. Knocking out *OsNAC006* using the CRISPR-Cas9 system results in significant drought and heat sensitivity in rice [188]. In conclusion, these transcription factors orchestrate rice’s tolerance to drought stress by modulating various gene expressions and initiating complex signaling pathways. Further exploration of these transcription factors’ specific mechanisms and interactions could yield new strategies and avenues for enhancing rice drought resistance breeding.

### 7.2. Role of miRNA in Rice Drought Stress Regulation

The regulation of miRNA in rice drought stress is a prominent area of research in plant biology. miRNAs assist plants in coping with environmental stresses such as drought by regulating gene expression, thereby enhancing their survival and productivity [189]. Research indicates that various miRNAs are significantly upregulated or downregulated under drought stress [190], playing critical roles in modulating rice’s response to such conditions. For instance, studies have shown that miRNAs like *miR159f*, *miR1871*, *miR398b*, and *miR408-3p* are upregulated in drought-tolerant varieties and downregulated in sensitive ones [191]. The expression patterns of specific miRNAs in different varieties and tissues reveal their essential functions in drought response [192]. miRNAs manage multiple signaling pathways to address drought stress, including the abscisic acid (ABA) signaling pathway, calcium signaling pathway, detoxification mechanisms, and lateral root formation, among others [193]. For example, the *miR159-MYB* module and *miR169-NFYA* module are involved in ABA-dependent signaling pathways, while *miR156-SPL* and *miR393-TIR1* participate in ABA-independent pathways [194]. *miR190* also plays a vital role in ABA-dependent drought tolerance. Under drought stress, miRNAs regulate physiological and biochemical responses by targeting specific transcription factors and functional protein-coding genes, e.g., *ZmmiR190* targets the *ZmCRP04* gene to regulate ABA-dependent drought tolerance [195]. miR408 regulates copper protein-encoding genes like plantacyanin, laccase, and copper-zinc superoxide dismutase (Cu/Zn SODs), which are significantly expressed during drought stress [196]. The expression patterns of miRNAs differ significantly between roots and shoots, indicating potential differential regulatory roles in various tissues [197]. For example, *miR169g* is significantly upregulated in rice roots, while its regulation in stems is minimal, suggesting a specific role for this miRNA in particular tissues in response to drought [198]. In summary, miRNAs play a critical role in rice drought stress response. They regulate multiple signaling pathways and target genes, helping plants adapt to drought conditions. Future research could further explore the specific functions and regulatory mechanisms of these miRNAs to enhance crop drought resistance through genetic engineering methods.

## 8. Breeding Drought-Resistant Rice Varieties

### 8.1. Traditional Breeding Methods

Drought stress significantly reduces rice yields, disrupting growth cycles and affecting physiological and biochemical processes. Traditional breeding approaches to enhance drought tolerance in rice primarily rely on selection and hybridization techniques such as pedigree selection, recurrent selection, and backcrossing. These methods involve screening and evaluating drought-tolerant traits while introducing and stabilizing beneficial genes [199].

Key traits targeted in breeding include root characteristics (e.g., deep and robust root systems), osmotic adjustment capacity, shorter growth durations, and smaller leaf areas [200]. A deep and well-developed root system helps rice access water from deeper soil layers, improving drought resilience [201]. Rice with strong osmotic adjustment capacity can maintain cellular water balance under drought stress, minimizing damage [202]. Varieties with shorter growth periods and smaller leaf areas demonstrate enhanced drought tolerance under water-limited conditions [203]. For instance, applying melatonin can increase drought resistance by enhancing antioxidant enzyme activity, osmotic adjustment, relative water content, and the root-to-shoot ratio [204].

Furthermore, research shows that manganese ferrite nanomaterials significantly improve drought tolerance by enhancing biomass production, photosynthesis, nutrient uptake, and polysaccharide levels, and activating drought-responsive genes [205].

Despite the successes of traditional breeding, significant challenges remain. Low heritability of drought tolerance traits slows the selection process and increases costs, while environmental variability reduces the reproducibility of selection outcomes [206]. To address these issues, breeders have adopted techniques such as managed drought stress and mutagenesis. Applying drought stress at specific growth stages can enhance the heritability of yield traits, while methods like radiation mutagenesis introduce drought-tolerance genes to produce more resilient rice varieties [199,207]. For example, the *ddt1* mutant, developed through EMS mutagenesis, exhibits enhanced drought resistance with higher survival rates, reduced water loss, and faster stomatal closure under drought conditions [208].

### 8.2. Modern Molecular Breeding Techniques

Recent advancements in gene-editing technologies, especially the CRISPR/Cas9 system, have revolutionized the development of drought-resistant rice varieties. CRISPR/Cas9 enables precise editing of target genes, allowing researchers to study gene functions and create mutants with improved traits. This technology efficiently produces specific, heritable genomic edits in rice, shortening breeding cycles and facilitating the development of drought-resistant varieties. For instance, editing the *OsSAP* gene revealed its role as a positive regulator of drought tolerance [209].

Knocking out the *RRS1* gene results in enhanced drought resistance by promoting water uptake and improving water use efficiency [210]. Similarly, *OsPYL9*-edited mutants show higher ABA accumulation, antioxidant activity, survival rates, and yield under drought conditions [86] (Table 1). Mutants of the *OsERA1* gene demonstrate increased ABA sensitivity and improved drought tolerance [211,212]. Additionally, *SRL1* and *SRL2* mutants generated through CRISPR/Cas9 exhibit higher survival rates and antioxidant enzyme activities under drought stress [213].

Gene stacking techniques, such as co-transformation or re-transformation, further enhance drought tolerance by combining multiple drought-related genes into a single locus. For instance, integrating genes associated with cellular detoxification, osmolyte accumulation, and antioxidant mechanisms improves both drought and salt tolerance. This approach surpasses traditional breeding or single-gene transformation by ensuring co-inheritance of the desired traits across generations [214].

Genomics-assisted breeding has also shown significant potential for developing drought-resistant rice varieties [215]. Tools such as marker-assisted selection (MAS), genomic selection, and QTL mapping and pyramiding allow for more efficient development of high-yielding, drought-tolerant rice [216]. MAS enables the integration of drought-tolerance QTLs into elite cultivars [214]. For example, combining QTLs such as *qDTY1.1*, *qDTY3.1*, and *qDTY12.1* with disease resistance genes like *Xa4*, *xa5*, *xa13*, and *Xa21* has led to the development of rice varieties resistant to both drought and diseases [217].

By utilizing MAS and QTL pyramiding, researchers have successfully developed drought- and disease-resistant rice varieties [218]. Through MAS and genomic selection, QTLs for drought tolerance have been introduced into the rice variety Hanhui3, significantly enhancing its drought and disease resistance [219].

Transgenic approaches are also widely used to breed drought-tolerant varieties. Some transgenic rice lines demonstrate significant drought resistance under field conditions without yield penalties. For instance, rice overexpressing the *OsHMGB707* gene enhances drought tolerance by promoting the expression of stress-responsive genes [220]. Meanwhile, RNAi silencing of the *OsMYB1R1* gene results in rice plants with higher survival rates and drought tolerance [221].

The integration of these advanced methods facilitates the development of high-yielding, drought-tolerant rice varieties, providing critical support for global food security and sustainable agricultural development.

**Table 1:** Drought resistance breeding genes.

Gene	Gene ID	Breeding Methods	Positive/Negative Regulation	References
*OsPYL9*	LOC_Os06g33690	Crispr Cas9	−	[86]
*OsPPR035/* *OsPPR406*	LOC_Os01g46230/LOC_Os10g30760	Crispr Cas9	−	[222]
*OsNAC092*	LOC_Os06g46270	Crispr Cas9	−	[223]
*OsDST*	LOC_Os03g57240	Crispr Cas9	−	[224]
*OsqRT9*	LOC_Os09g28210	Crispr Cas9	+	[225]
*OsOLP1*	LOC_Os08g07840	Over Expression	+	[226]
*OsPAPH1*	LOC_Os01g58640	Over Expression	+	[227]
*OsWRKY97*	LOC_Os01g09080	Over Expression	+	[24]
*OsHMGB707*	LOC_Os04g47690	Over Expression	+	[220]
*OsSYT-5*	LOC_Os07g22640	RNAi	−	[228]
*OsPYL6*	LOC_Os05g39580	RNAi	+	[85]

## 9. Conclusions and Prospects

This article provides a comprehensive review of recent advancements in the study of signaling pathways that regulate drought stress in rice. By examining the effects of drought stress on rice from morphological, physiological, biochemical, and molecular perspectives, this review delves into how rice adapts and regulates its mechanisms to contend with drought conditions. Research indicates that rice adapts to environmental changes through various strategies, including modifying root architecture, curling leaves, regulating stomata, and enhancing water use efficiency. The initial perception and transmission of drought signals, the pivotal role of hormonal signals such as abscisic acid (ABA), and the regulation of downstream signaling networks like the MAPK and Ca^2+^ pathways, are all crucial for the drought resistance mechanisms in rice. Furthermore, the identification and functional analysis of drought-related genes, coupled with the potential application of gene-editing technologies in drought research, pave new pathways for future studies. Therefore, this review offers valuable insights and guidance for future research in drought-resistant rice breeding and agricultural production.

Despite notable advancements in our understanding of drought stress signaling regulation in rice, several critical challenges remain. First, there is a need to further elucidate the mechanisms of drought signal perception and initial transmission to better understand how plants rapidly respond to drought stress. Second, while ABA plays a significant role in drought responses, the interactions and regulatory networks of other plant hormones like ethylene, jasmonic acid, and gibberellins in drought conditions require deeper investigation. Additionally, a detailed study of the regulation mechanisms and interactions within downstream signaling pathways, such as MAPK and Ca^2+^, is also crucial.

With the rapid advancement of genome-editing technologies, such as CRISPR/Cas9, there is potential to enhance rice’s drought resistance by precisely modifying key genes. However, addressing the safety assessments of genetically modified organisms and their environmental impacts remains crucial for practical applications. Future research should integrate laboratory studies with field trials to advance the practical application of drought-resistance breeding techniques. Overall, understanding the signaling pathways that regulate rice drought stress is vital for addressing global climate change and ensuring food security. By integrating biotechnology, genetic breeding, and agricultural management strategies, we can provide new perspectives and solutions for drought resistance research in rice, thereby enhancing its productivity and stability under drought-prone conditions.

## Data Availability

All data generated and analyzed during this study are included in this published manuscript.
